# The Soil Bacterium *Methylococcus capsulatus* Bath Interacts with Human Dendritic Cells to Modulate Immune Function

**DOI:** 10.3389/fmicb.2017.00320

**Published:** 2017-02-28

**Authors:** Stine Indrelid, Charlotte Kleiveland, René Holst, Morten Jacobsen, Tor Lea

**Affiliations:** ^1^Research and Innovation, Østfold Hospital TrustKalnes, Norway; ^2^Department of Chemistry, Biotechnology and Food Science, Norwegian University of Life SciencesAas, Norway

**Keywords:** dendritic cells (DC), old friends hypothesis, immune modulation, environmental bacteria, DC activation, T cell polarization, immunobiotics, soil bacteria

## Abstract

The prevalence of inflammatory bowel disease (IBD) has increased in Western countries during the course of the twentieth century, and is evolving to be a global disease. Recently we showed that a bacterial meal of a non-commensal, non-pathogenic methanotrophic soil bacterium, *Methylococcus capsulatus* Bath prevents experimentally induced colitis in a murine model of IBD. The mechanism behind the effect has this far not been identified. Here, for the first time we show that *M. capsulatus*, a soil bacterium adheres specifically to human dendritic cells, influencing DC maturation, cytokine production, and subsequent T cell activation, proliferation and differentiation. We characterize the immune modulatory properties of *M. capsulatus* and compare its immunological properties to those of another Gram-negative gammaproteobacterium, the commensal *Escherichia coli* K12, and the immune modulatory Gram-positive probiotic bacterium, *Lactobacillus rhamnosus* GG *in vitro*. *M. capsulatus* induces intermediate phenotypic and functional DC maturation. In a mixed lymphocyte reaction *M. capsulatus*-primed monocyte-derived dendritic cells (MoDCs) enhance T cell expression of CD25, the γ-chain of the high affinity IL-2 receptor, supports cell proliferation, and induce a T cell cytokine profile different from both *E. coli* K12 and *Lactobacillus rhamnosus* GG. *M. capsulatus* Bath thus interacts specifically with MoDC, affecting MoDC maturation, cytokine profile, and subsequent MoDC directed T cell polarization.

## Importance

There has been a growing interest in probiotics for treating both IBD, allergies, and autoimmune diseases, and considerable effort has been invested in identifying novel probiotics aimed for treating immune pathologies. Typically, candidate probiotic bacteria has been of human or animal origin, and a host-associated lifestyle is assumed to be a prerequisite for developing immune-regulatory functions. Here we describe immune modulatory functions of a non-commensal soil bacterium previously shown to exhibit anti-inflammatory effects in a murine colitis model pointing to environmental bacteria as a new and untapped source of bacteria for modulating immune responsiveness.

## Introduction

Although microbes are associated with all epithelial surfaces of animal hosts, the highest number, and most diverse microbial populations are found in the intestines. Some 10–100 trillion microbes colonizes the human gastrointestinal tract, with the highest numbers present in the colon (Turnbaugh et al., 2007). The physiology of these microbes and their hosts is closely connected and mutually regulated (Brown et al., 2013). The host shapes the composition of the intestinal microbiota at species and community levels by supplying nutrients and by producing antimicrobial peptides. The human microbiota in return, adds to the metabolic, and biochemical activities of the host and play essential roles in the development and differentiation of the host intestinal epithelium, the immune system, and in the maintenance of mucosal homeostasis (Nicholson et al., 2012; Sommer and Backhed, 2013).

Only a single layer of epithelial cells separates the luminal contents and microbial community from underlying tissues, and the epithelial barrier therefore provides a possible entry point for opportunistic pathogens into the body. The host must maintain a mutualistic relationship with the commensal microbiome, while retaining protective responsiveness against pathogenic bacteria. To achieve this it must preserve epithelial integrity and regulate pro- and anti-inflammatory signaling, in an appropriate manner. Homeostasis is maintained through continuous and dynamic interactions and communication between the intestinal microbiota, the epithelium, and immune cells in the intestinal mucosa.

The regulatory interactions that exist between multicellular organisms and the microbial world are not necessarily limited to those between commensals and their hosts. The increasing prevalence of inflammatory bowel disease and autoimmune diseases in the western world has been associated with reduced exposure to helminths and environmental microorganisms from soil, water, and fermenting vegetables (Rook, 2007). The “hygiene hypothesis” was forwarded as a result of studies coherent with the idea that postnatal infections may be protective against allergy later in life, and that such protection may be lost in the presence of modern hygiene (Strachan, 1989, 2000). The related “old-friend hypothesis” explains the striking increase in chronic inflammatory disorders as largely being due to reduced contact with microorganisms that we have coevolved with, and therefore depend on, for proper immune development and regulation (Rook, 2010). In this context both pathogenic bacteria, the commensal microbiota, pseudo-commensals, and even the environmental microbiota may be essential regulatory components of the mammalian immune system. An increased mechanistic understanding of how such microbes and microbial products affect immune homeostasis may form a basis for developing novel tools for modulating immune responses in chronic inflammatory disorders.

Recently we demonstrated that a bacterial meal of the Gram-negative soil bacterium, *Methylococcus capsulatus* Bath, ameliorates dextran sulfate sodium (DSS) induced colitis in mice (Kleiveland et al., 2013). The study points to a potential for non-commensal environmental bacteria in protecting against experimental colitis in mammals, but the mechanisms behind these effects have not been identified. Both live and heat-killed probiotic bacteria have previously been shown to protect against experimental colitis (Mileti et al., 2009; Sang et al., 2014; Toumi et al., 2014; Souza et al., 2016). Proposed modes of action include competitive pathogen exclusion, production of antimicrobial substances, gut flora modulation, modulatory effects on epithelial barrier integrity, regulatory effects on innate, and adaptive immunity and effects on epithelial development and survival (Bermudez-Brito et al., 2012). However, direct effects on dendritic cells (DCs) with subsequent effects on cytokine production and T cell development is expected to be a common mode of action for those probiotic strains able to modulate adaptive immunity (Bienenstock et al., 2013).

DCs are professional antigen presenting cells that play a key role in both innate and adaptive immune responses (Steinman, 2012). Intestinal DCs expresses pattern recognizing receptors (PRRs) to recognize various microbial structures and can distinguish between microbe-associated molecular patterns (MAMPs) of even closely related organisms to initiate specific and appropriate response. The capacity of DCs to activate naïve T cells inducing T cell expansion and polarization, position DCs as critical mediators of host immune tolerance, and inflammatory responses (Mann et al., 2013).

The dietary inclusion of *M capsulatus* Bath in DSS-colitis model affected the intestinal epithelium through increased cell proliferation and mucin production, suggesting beneficial effects on gut barrier function. However, direct effects on cells of the immune system was not evaluated in that study. Here, for the first time, we show that *M. capsulatus* Bath, a non-commensal environmental bacterium, specifically and strongly adheres to murine and human DCs, an immune cell type central in regulating both innate and adaptive immunity. We compare the immune modulatory effects of *M. capsulatus* Bath to those of the Gram-negative commensal *Escherichia coli* K12, a non-pathogenic *E. coli* strain originally isolated from stool of a diphtheria patient (Agency USEP, 1997), and the well characterized Gram-positive probiotic bacterium *Lactobacillus rhamnosus* GG. The interaction between DC and *M. capsulatus* leads to functional activation of the DCs, affects DC cytokine profile, improves T cell activation, and proliferation and drive T effector cell polarization *in vitro*.

## Materials and methods

### Bacterial strains and culture conditions

*M. capsulatus* strain (Bath) (NCIMB 11132, Aberdeen, UK) were grown in nitrate mineral salts medium (Whittenbury et al., 1970) with a head-space of 75% air, 23.75% CH_4_, and 1.25% CO_2_ at 45°C and 200 rpm. *E. coli* strain K12 was kindly provided by Department of Bacteriology, the Norwegian Veterinary Institute, Norway. *E. coli* K12 (Blattner et al., 1997) was grown in LB medium (Oxoid Ltd., Basingstoke, United Kingdom) at 37°C and 200 rpm. *L. rhamnosus* GG was grown in MRS medium (Oxoid Ltd.) anaerobically at 37°C without shaking.

### Cells and culture conditions

Human erythrocyte- and plasma depleted blood were obtained from healthy volunteers from Ostfold Hospital Trust, Fredrikstad, Norway in accordance with institutional ethical guidelines and with approval from the Regional Committee of Medical and Health Research Ethics with written informed consent from all subjects. All subjects gave written informed consent in accordance with the Declaration of Helsinki. Peripheral blood mononuclear cells (PBMCs) were isolated by density gradient centrifugation on a Lymphoprep gradient (Fresenius Kabi). Human T cells were isolated from PBMCs by negative selection using Dynabeads Untouched Human T Cells Kit (Thermo Fisher). CD14^+^ cells were isolated by positive selection using human CD14 MicroBeads (Miltenyi Biotech). To develop immature monocyte-derived dendritic cells (MoDCs) CD14^+^ cells were cultivated for 6 days in RPMI 1640 medium supplemented with 10% heat inactivated fetal calf serum and 25 μg/ml gentamicin sulfate (Lonza), 1 mM sodium pyruvate and 100 μM non-essential amino acids (both from PAA Laboratories), 25 ng/ml interleukin 4 and 50 ng/ml granulocyte macrophage colony stimulating factor (both from ImmunoTools).

### Bacterial stimulation, cytokine analysis, and immune phenotyping of MoDCs

For immune phenotyping and DC cytokine analysis MoDCs were primed for 24 h by bacteria in a ratio of 1:10 (MoDC: bacteria) or by a maturation cocktail of 15 ng/ml TNF-α (ImmunoTools), 100 ng/ml LPS and 5 μg/ml PGE2 (Sigma-Aldrich). Culture supernatants were harvested and stored at −20°C then analyzed for cytokines by ProcartaPlex Multiplex immunoassay (eBioscience). TGF-β and IL-6 was measured by ELISA kits (eBiosciences and PeproTech respectively). MoDCs were also harvested and stained using PE-conjugated mouse anti-human CD80 antibodies, PE-Cy5 conjugated mouse anti-human CD83, and PE-Cy5 conjugated mouse anti-human CD40 (all from BD Biosciences). For viability testing cells were stained by 1μg/ml PI and analyzed by flow cytometry.

### DC-T cell co-cultures for cytokine analysis and immunophenotyping

To induce antigen specific T cell responses a modified mixed leukocyte culture system (MLC) were used with MoDC as stimulator cells and purified peripheral blood T cells as responder cells. MoDCs, either unprimed or primed by UV-inactivated bacteria in a ratio of 1:100 (MoDC:bacteria) for 24 h, were co-incubated with allogeneic T cells from two different donors (1:10 ratio between MoDCs and T cells). For cytokine analysis cells were grown in 48 well plates. After 5 days culture supernatants were harvested and T cells phenotyped by flow cytometry using FITC-conjugated anti-human CD4 and APC-conjugated anti-CD25 (both from Miltenyi Biotech). Fluorescence was detected by a MACSQuant flowcytometer and analyzed using the MACSQuantify software (both from Miltenyi Biotech). Cytokine concentrations in culture supernatant were measured by ProcartaPlex Multiplex immunoassay (eBioscience).

### T cell proliferation assay

MoDCs were primed for 24 h with UV-inactivated *M. capsulatus* 1:100 (DC:bacteria) in Nunc™ UpCell™ plates (Thermo Fisher). After 24 of stimulation the MoDCs were harvested, washed and co-incubated with allogenic human T cells in 96-well plates in a ratio of 1:10 (DC:T cells). Next day recombinant human IL-2 was added to each well to a final concentration of 10 U/ml. After 96 h of co-culture cells were pulsed by [^3^H]-thymidine (1 μCi, Perkin Elmer) for 18.5 h. Cells were harvested onto glass-fiber filters and incorporated thymidine determined by liquid scintillation counting using a TopCount NXT™ Luminometer (Packard BioScience Company).

### Scanning electron microscopy (SEM)

Immature MoDCs were co-cultivated with *M. capsulatus* Bath in 1:100 ratio (cells:bacteria) in medium free of antibiotics for 2–4 h in a humified atmosphere with 5% CO_2_. Cells were washed twice by phosphate buffered saline (PAA Laboratories), fixed with 4% PFA and 2.5% glutaraldehyde (1:1) for 20 min at room temperature. Cells were washed again, dehydrated in a graded ethanol series and dried using a critical point dehydrator (CPD030 BalTec). Samples were coated with ~500 Å Pt in a sputter coater (Polaron SC7640, Quorum technologies) and analyzed using an EVO-50 Zeiss microscope (Carl Zeiss AG).

### Confocal imaging

Immature MoDCs were generated from CD14^+^ monocytes as described above. 1 × 10^8^/ml *M. capsulatus* Bath were stained by 10 μM CFSE in PBS. CFSE-stained bacteria were co-incubated with immature MoDCs in a ratio of 1: 100 cells-bacteria. Cells were washed, fixated in PBS with 1% formalin then washed twice before coverslip was mounted on object slide with ProLong Diamond Antifade Mountant with DAPI (Thermo Fisher Scientific). Samples were scanned under a Zeiss LSM510 META confocal microscope (Carl Zeiss). Confocal stacks were acquired with z-spacing of 0.2 μm.

### Statistical analysis

Data were sampled in hierarchical structure, with multiple measurements per individual. This violates the assumption of independent measurements underlying ANOVA and conventional linear regression. This issue was remedied by analyzing the data using a mixed effects linear model, in which each individual acted as a random effect. Box-Cox analyses were used for finding suitable normalizing transformations. Data were analyzed on the log-scale and subsequently back-transformed for interpretation. All analyses were controlled by residual plots and Shapiro-Wilks test for normality.

## Results

### *M. capsulatus* bath adheres specifically to MoDC

**Figure 1:**
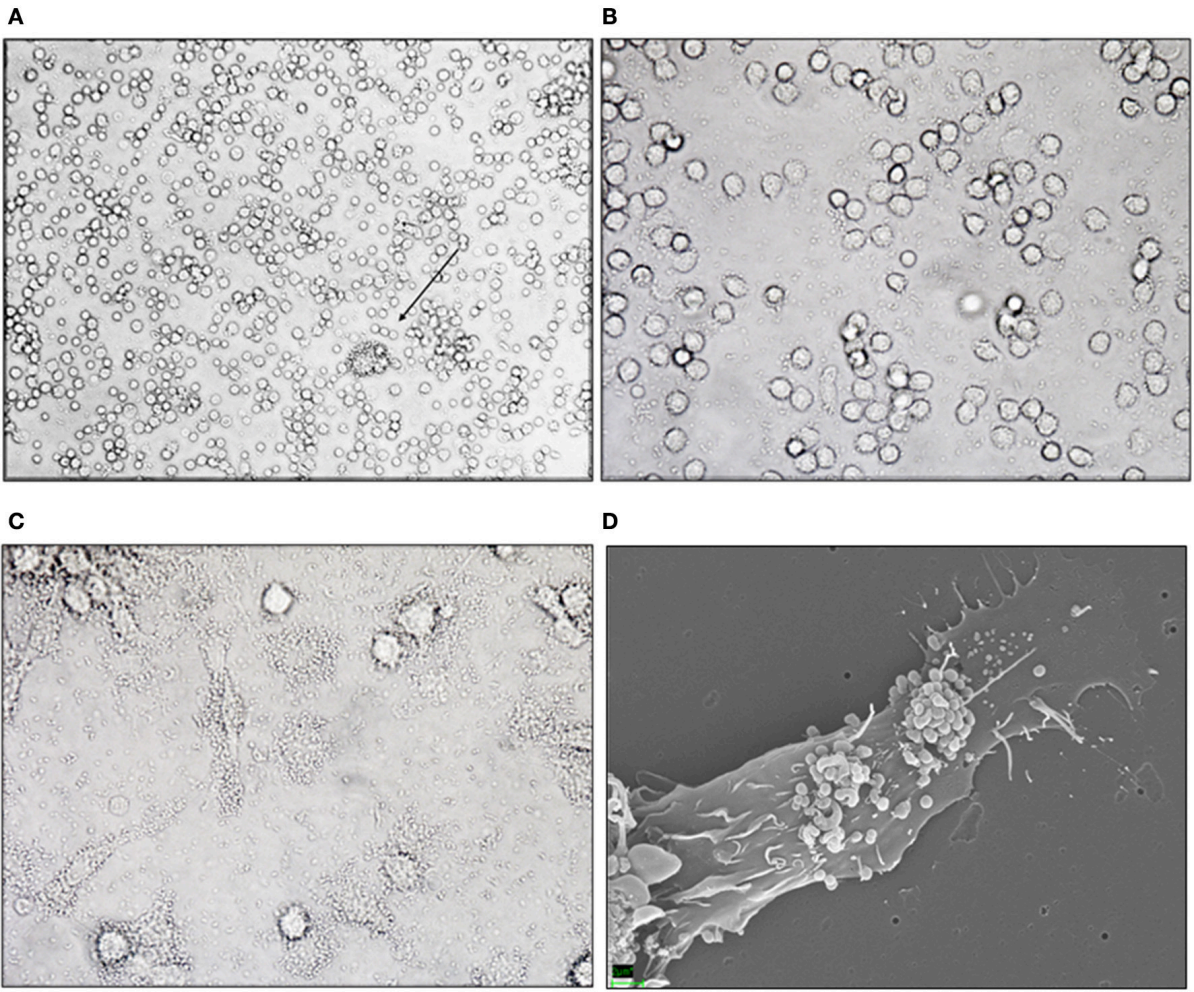
*****M. capsulatus*** Bath interacts specifically with human MoDCs. (A–C)** Light microscopy image of *M. capsulatus* Bath co-incubated (1:100 cells:bacteria) with human PBMC, CD14^+^ monocytes, or monocyte-derived dendritic cells without washing. *M. capsulatus* Bath clusters around low frequency-cells in PBMC **(C)** (arrow), but not CD14^+^ monocytes **(B)**. In co-culture with MoDCs bacteria cluster around a majority of cells **(C)**. **(D)** SEM electrograph showing *M. capsulatus* Bath adhering to human MoDCs after 3 h co-incubation.

A bacterial protein preparation of *M. capsulatus* Bath was previously found to have anti-inflammatory effects in a murine model of colitis (Kleiveland et al., 2013). When studying possible immune modulatory effects on immune cells, we observed that bacteria clustered around a small subset of cells in peripheral blood mononuclear cell preparations (Figure 1A). The appearance and low frequency of the target cells were consistent with the size and expected frequency of DCs among PBMCs. To determine whether the target cells were in fact DCs we incubated *M. capsulatus* Bath with CD14^+^ monocytes or MoDCs generated from CD14^+^ monocytes in the presence of IL-4 and GM-CSF. *M. capsulatus* did not bind to CD14^+^ monocytes (Figure 1B), but quickly associated with dendritic cells (Figure 1C). The interaction between *M. capsulatus* Bath and MoDCs was further visualized by scanning electron microscopy (SEM) showing *M. capsulatus* Bath in large clusters on MoDCs after 3 h of co-incubation, even after several washes with PBS (Figure 1D).

**Figure 2:**
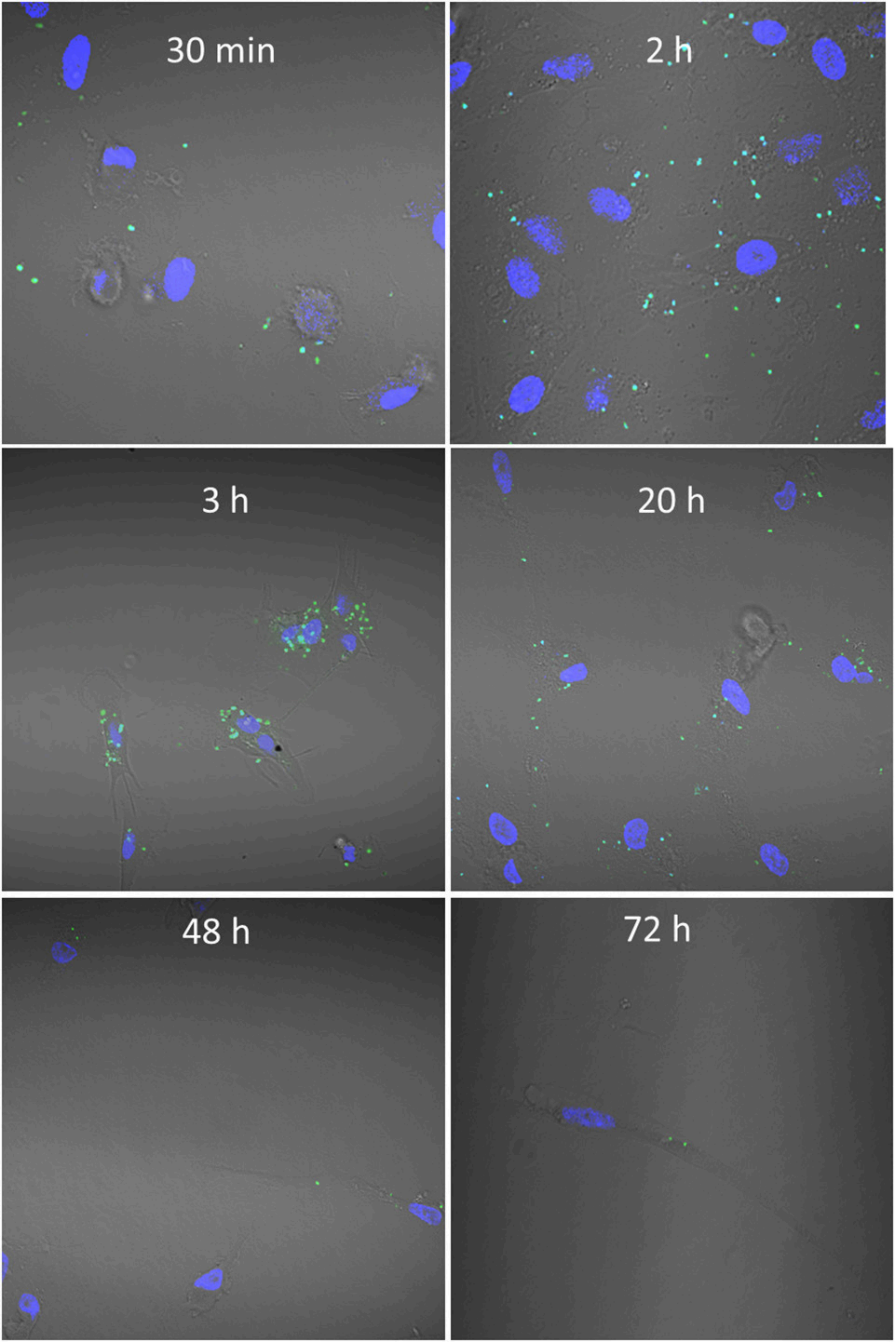
*****Methylococcus capsulatus***—DC interaction kinetics**. Figure shows CFSE labeled *M. capsulatus* Bath (green) co-incubated with human MoDCs for 30 min to 72 h. MoDC nuclei were counterstained with DAPI (blue) to aid visualization and interactions were visualized by confocal microscopy.

To study binding kinetics we co-incubated CFSE-stained bacteria with MoDCs. Cells were counterstained with DAPI and confocal microscopy was used to visualize interactions over time (Figure 2). *M. capsulatus* Bath were found in scattered association with cells after just 30 min of co-incubation, and after 2 h a large number of bacteria associated with most cells. Strikingly, after around 3 h of co-incubation *M. capsulatus* were typically found to cluster around the nucleus of the MoDCs. A large number of bacteria could be seen associated with cells up to 20 h after co-incubation. At 48 h bacteria were cleared from most cells although a few intact bacteria was found associated with cells up to 72 h after co-incubation (Figure 2).

### *M. capsulatus* bath induces phenotypic and functional maturation of MoDCs

**Figure 3:**
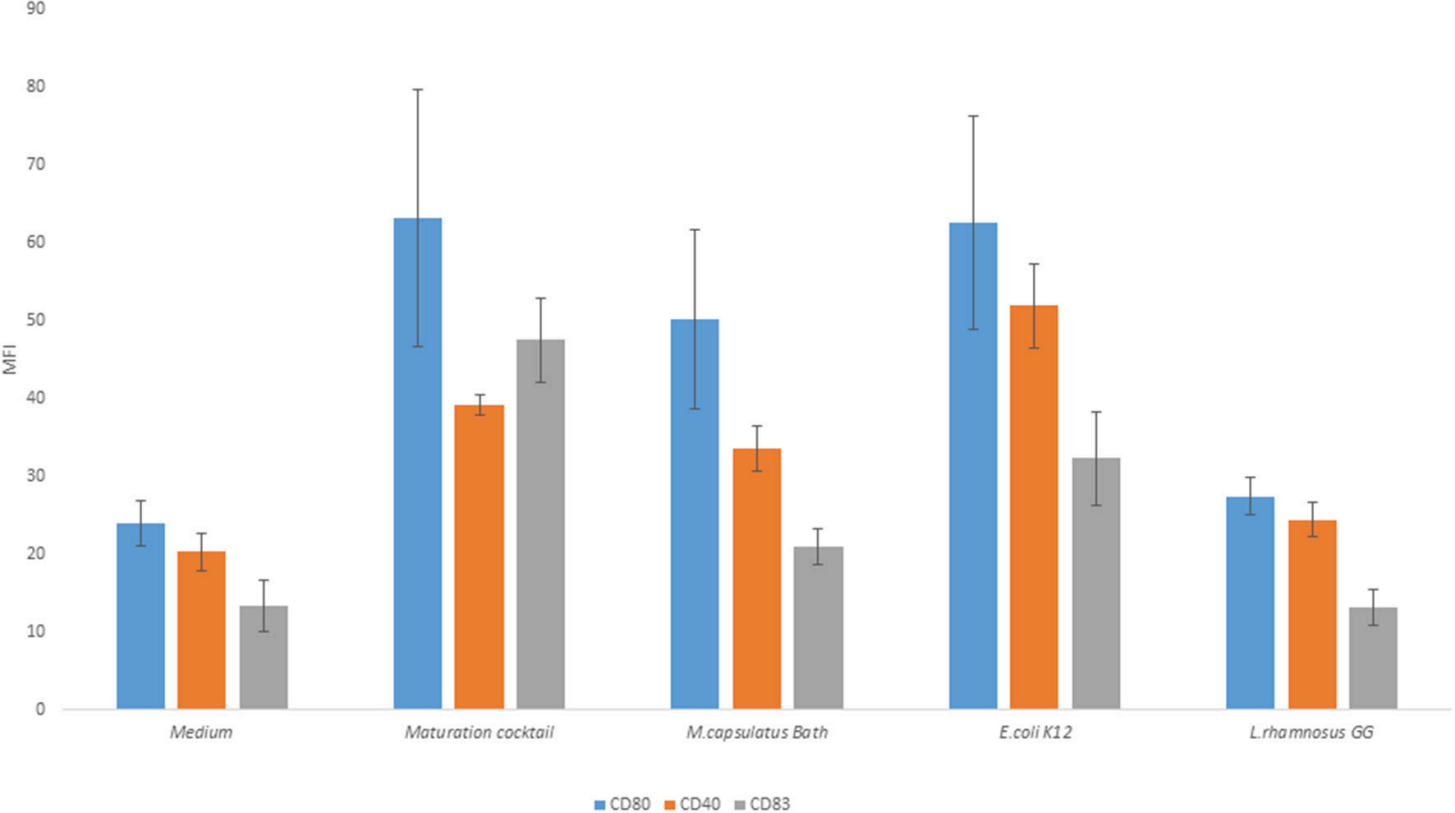
*****M. capsulatus*** Bath and ***E. coli*** K12 induce maturation of MoDCs**. Human MoDCs were either activated by a maturation cocktail of TNF-α, PGE2, and LPS or co-incubated with bacteria (*M. capsulatus* Bath, *E. coli* K12, or *L. rhamnosus* GG) for 24 h. Cells were stained for CD80, CD83, and CD40 and analyzed by flow cytometry in this figure. Median fluorescence intensity (MFI) is reported. Error bars indicate standard error on median fluorescence intensity values from 6 different donors.

Upon microbial stimulation, DCs undergo a program of maturation that endows them with capacity to activate naïve T cells, induce T cell expansion, and to polarize T cells toward effector subpopulations appropriate to the stimulus encountered. Mature DCs are characterized by expression of co-stimulatory molecules required for efficient T cell activation. We compared the ability of *M. capsulatus* Bath, Gram-positive, and Gram-negative control bacteria to induce MoDC activation as assessed by expression of costimulatory molecules like CD40, CD80, and CD83. MoDCs, either left unprimed or co-incubated with bacteria (*M. capsulatus* Bath, *L. rhamnosus* GG, or *E. coli* K12) were stained for co-stimulatory molecules and maturation markers and analyzed by flow cytometry (Figure 3). Cells activated by a maturation cocktail containing TNF-α, LPS, and PGE_2_ were used as a positive control. The maturation cocktail, *E. coli* K12, and *M. capsulatus* Bath induced upregulation of CD40, CD83, and CD80 in immature MoDCs. *E. coli* K12 was found to represent the most potent bacterial stimulus for inducing a mature DC phenotype compared to unprimed control cells, and induced expression of all activation markers. *M. capsulatus* Bath showed intermediate ability to induce MoDC maturation and elicited CD40 and CD80 expressions comparable to positive control, but a lower expression of CD83 compared to *E. coli* and the maturation cocktail (Figure 3). *L. rhamnosus* GG was a weak inducer of MoDC maturation, and produced a phenotype similar to unprimed cells. Cell viability, determined by PI staining, was similar between treatments suggesting that none of the strains asserted toxic effects on MoDCs (Data not shown).

### MoDCs respond to bacterial stimulation by eliciting distinct cytokine profiles

**Figure 4:**
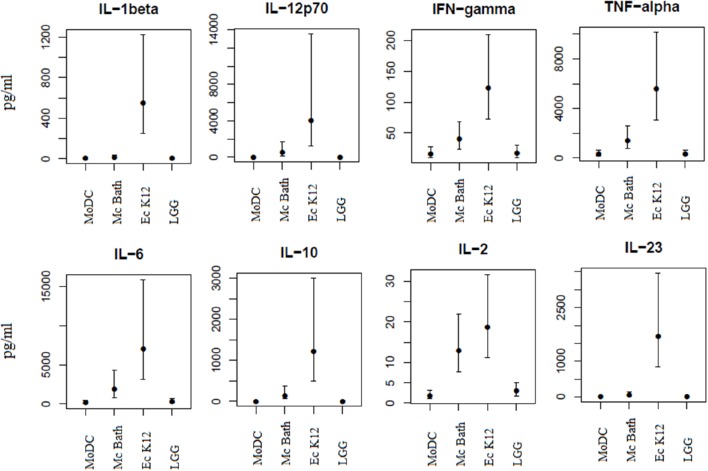
**MoDCs produce distinct cytokine profiles in response to different bacteria**. MoDCs were incubated with bacteria *(M. capsulatus* Bath, *E. coli* K12, *L. rhamnosus* GG) for 24 h in a ration of 1:10 (DC: bacteria). Culture supernatants were collected and analyzed for cytokines by multiplex immunoassay or ELISA. Cytokine concentrations are given in picogram/milliliter. Bars represents 95% confidence intervals on cytokine concentrations from 4 different donors.

Depending on the nature of the stimuli, functionally mature DCs release cytokines promoting differentiation of naïve T cells into specific effector cell subsets. Since *M. capsulatus* and *E. coli* induced phenotypic maturation of MoDCs we wanted to see whether the bacteria also resulted in functional maturation characterized by cytokine release. Multiplex immunoassay and enzyme-linked immunosorbent assay (ELISA) was used to measure select cytokines in culture supernatants of MoDCs co-cultivated with bacteria for 24 h (Figure 4). In general, and in accordance with the observed phenotypic activation of MoDC, *E. coli* K12 was the most potent inducer of cytokine production, and resulted in increased release of IL-1β, IL-12p70, IL-10, TNF-α, IL-2, IL-23, IFN-γ, and IL-6 compared to unprimed control. *L. rhamnosus* GG in comparison was the least potent inducer of cytokine production in MoDCs of the three tested bacteria (Figure 4). Incubation with *M. capsulatus* Bath resulted in intermediate levels of cytokines. Similar to *E. coli*-primed MoDCs, incubation with *M. capsulatus* enhanced the production of IL-12p70, IL-10, TNF-α, IL-2, and IL-23 compared to unprimed MoDCs. However, *M. capsulatus* treatment in general resulted in lower cytokine levels than *E. coli* K12. *M. capsulatus*-primed cells produced substantially less IL-1β, IL-6, IL-10, IL-12p70, IL-23, and TNF-α than *E. coli*-primed cells, but the two bacteria induced similar levels of IL-2 from the MoDCs. TGF-β could not be detected in any of the co-cultures (data not shown). Thus, the interaction between *M. capsulatus* Bath and MoDCs resulted in both quantitative and qualitative differences in cytokine production compared to *E. coli* K12.

### *M. capsulatus* bath increases DC-induced T cell activation and proliferation

**Figure 5:**
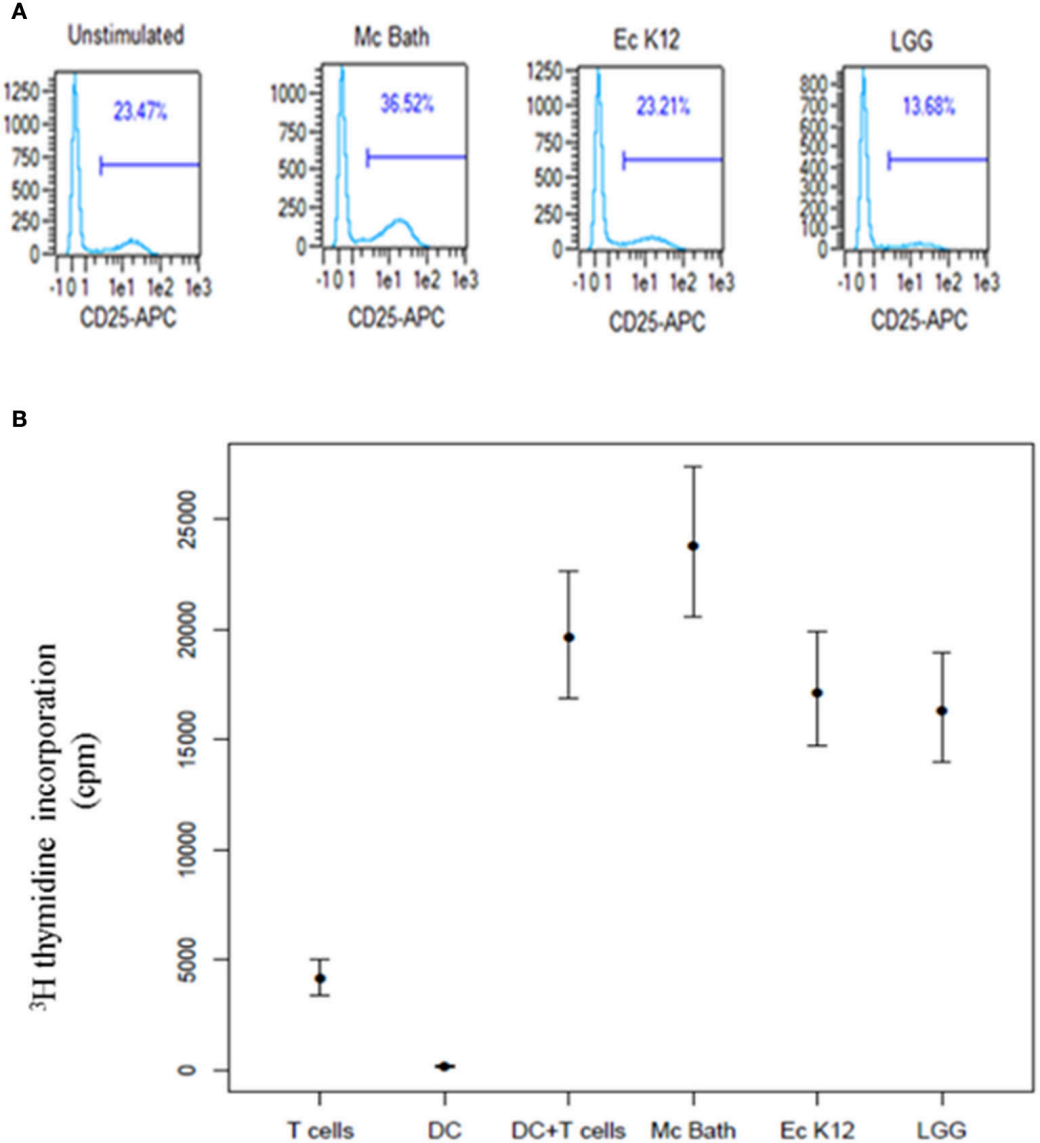
*****M. capsulatus*** primed MoDCs efficiently induce T cell activation and proliferation. (A)** Immature MoDCs were primed by UV inactivated bacteria for 24 h in a ratio of 1:100 (DC: bacteria). Primed MoDCs were co-incubated with allogenic T cells in the presence of IL-2. After 5 days of co-culture cells were harvested, stained for CD4 and CD25 surface protein and analyzed by flow cytometry. Cells were gated on CD4-FITC expression and the percentage of CD4^+^ cells expressing CD25 are shown. Plots represent results from 4 different MoDC/T cell donor combinations. **(B)** MoDCs primed by either UV-inactivated *M. capsulatus* Bath, *E. coli* K12, or *Lactobacillus rhamnosus* GG for 24 h were co-incubated with allogenic T cells from two different donors. After 96 h cells were pulsed by 1 μCi [^3^H] thymidine. Thymidine incorporation was determined by liquid scintillation counting 18.5 h after pulsing. The amount of incorporated thymidine is reported as counts per minute (cpm). Bars indicate 95% confidence interval on values from 5 different donor combinations.

Antigen recognition and a co-stimulatory signal through CD28 on T cells is required to induce functional T cell activation and clonal expansion. As the bacteria differently induced expression of DC co-stimulatory molecules, we examined the ability of bacteria-primed MoDCs to activate and induce proliferation in peripheral blood T cells. We co-incubated bacteria-primed MoDCs with allogenic T cells and measured expression of CD25 by flow cytometry. T cells co-cultivated with *M. capsulatus*-primed MoDCs expressed increased levels of CD25 compared to T cells cultivated with unprimed MoDCs or MoDCs primed by any of the control bacteria (Figure 5A). To test the ability of bacteria-treated MoDCs to induce proliferation in allogeneic T cells we measured DNA synthesis by [^3^H] thymidine incorporation. MoDCs activated by *M. capsulatus* were stronger supporters of T cell proliferation than MoDCs primed by any of the control bacteria (Figure 5B).

### MoDCs primed by different bacteria have different ability to drive T cell differentiation

**Figure 6:**
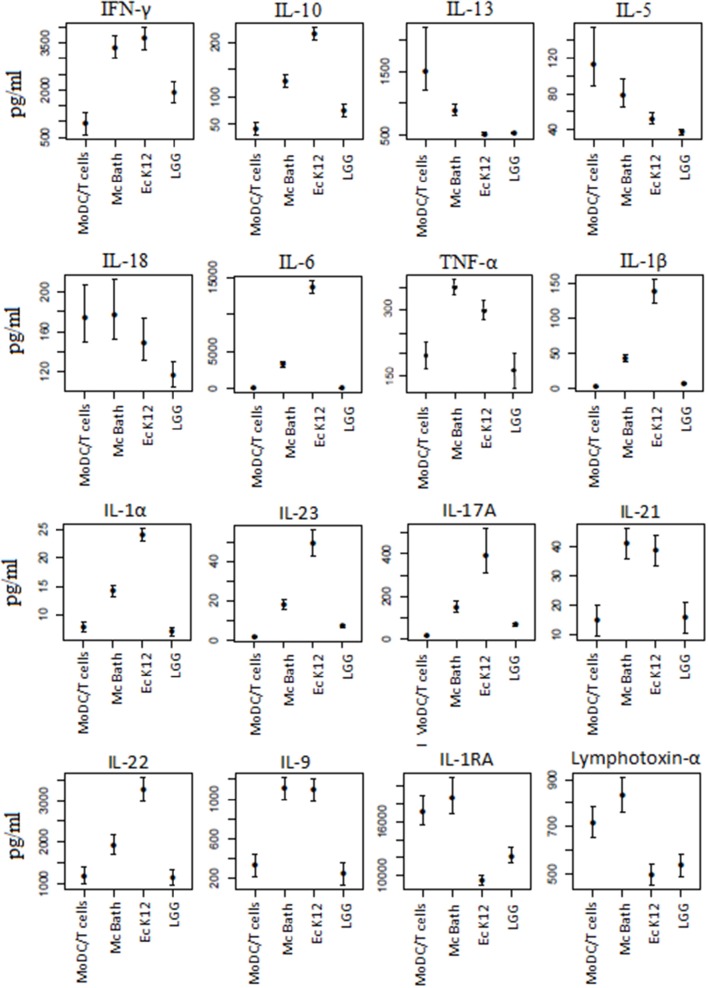
**Bacterial stimulation results in different effector T cell profiles**. Unprimed MoDCs or MoDCs primed by *M. capsulatus* or control bacteria were co-incubated with allogenic T cells for 5 days. Growth medium was collected and analyzed for cytokines by multiplex immunoassay. Bars represent 95% confidence intervals on cytokine concentrations from 4 donor combinations.

Cytokines produced by mature DCs contribute to drive T cell differentiation into specific effector cell subsets. In order to evaluate functional effects of bacteria-treated MoDCs on T cell polarization, activated MoDCs were co-incubated with allogeneic T cells. Culture medium was collected and analyzed for cytokines associated with different effector T cell subsets (Figure 6). MoDCs primed by any of the bacteria resulted in markedly reduced levels of typical Th2 cytokines like IL-5 and IL-13. All bacteria further resulted in increased release of the Th1 cytokine IFN-gamma and IL-10, an anti-inflammatory cytokine produced by several effector T cell lineages, compared to the basal level produced by T cells co-incubated with unprimed MoDCs.

Although all bacteria shifted T cells toward a Th1 rather that a Th2 phenotype, a major difference was found between Gram-negative *M. capsulatus* Bath and *E. coli* K12 on the one hand and Gram-positive *L. rhamnosus* GG on the other. Compared to T cells co-cultivated with unprimed MoDCs only *L. rhamnosus*-treated MoDCs resulted in significantly reduced release of IL-18, a proinflammatory cytokine that enhances innate immunity as well as Th1- and Th2-driven immune responses depending on cytokine milieu.

Conversely, only the Gram-negative bacteria *M. capsulatus* Bath and *E. coli* K12 gave significantly higher levels of the proinflammatory cytokines IL-6, TNF-α, IL-1β, and IL-1α. Both bacteria also increased IL-23, a cytokine linked to the generation and maintenance of Th17 cells, Th17 cytokines (IL-17A, IL-21, IL-22), Th22 cytokines (IL-22, TNF-α), and Th9 cytokines (IL-9 and IL-21).

Not all differences could be attributed to dissimilarities between Gram-positive vs. Gram-negative bacteria, however. No significant difference was found between *E. coli* and *M. capsulatus* in their ability to induce Th1, Th22, or Th9 cells, as evaluated by IFN-γ, TNF-α, IL-9, and IL-21, but compared to *E. coli, M. capsulatus* resulted in significantly less IL-23, Th17- associated cytokines IL-17A, and IL-22 as well as pro-inflammatory cytokines IL1-α, IL-1β, and IL-6 and the anti-inflammatory cytokine IL-10. Furthermore, reduction in Th2 cytokines IL-5 and IL-13 was lowest for *M. capsulatus* Bath primed co-cultures and *E. coli* and *L. rhamnosus*, but not *M. capsulatus* Bath, reduced IL-1RA and lymphotoxin-α levels in the cultures. *M. capsulatus* thus induces a T cells response functionally distinct from both *E. coli* K12 and *L. rhamnosus* GG.

## Discussion

Previous studies have described protective properties of probiotic bacteria, commensals, and their metabolites against experimental colitis in animal models (Pils et al., 2011; Qiu et al., 2013; Toumi et al., 2014; Souza et al., 2016). Although a connection between chronic intestinal inflammation and a reduced exposure to bacteria from soils and water has been made (Rook, 2007), few studies have focused on immune modulatory effects of non-commensal environmental bacteria. Here we show that a soil bacterium previously shown to reduce symptoms of chemically induced colitis in mice (Kleiveland et al., 2013) specifically targets human dendritic cells *in vitro*, affecting DC maturation, T cell activation, proliferation, and differentiation. *M. capsulatus* Bath was found to adhere specifically to human DCs. To our knowledge, this is the first report of an environmental bacterium to target mammalian DCs to modulate immune function.

The realization that a soil bacterium interacts specifically with human DCs raises some important questions. The significance of the commensal microbiome in health and disease is increasingly recognized, and there is a growing interest in probiotics within the scientific and public community. However, the role of environmental bacteria in immune regulation has been underappreciated for understandable reasons. It is not difficult to imagine that commensals living in close connection with humans are also closely connected to human physiology (Sommer and Backhed, 2013). Similarly, there is a long history of probiotics in fermented food associated with longevity and health. In a modern world of reduced microbial diversity it may be less intuitive to connect environmental bacteria to regulation of human health. However, as emphasized by the “old friends” hypothesis, mammals are evolutionary linked not only to commensals and probiotics, but also to ambient microbes in both soil and water (Rook, 2010).

Not only have mammals evolved from environmental bacteria, but the mammalian immune system has developed in the presence of such microbes. Throughout evolution some of these microbes may have taken on functions for us, some may relay signals necessary for immune development, while others, because of our long evolutionary association, are recognized by the immune system as harmless and have taken on regulatory roles (Rook et al., 2004). For example, chronic exposure to environmental LPS and other bacterial components present in farm dust may protect against asthmatic disease (Schuijs et al., 2015) possibly by reducing the overall reactivity of the immune system.

*M. capsulatus* Bath is an environmental bacterium that has been isolated from soil, water, sewage, mud, and lake sediments. It does not require a host to survive and may therefore face no obvious selection pressure to express immune modulatory molecules. Nevertheless, as discussed by Casadevall and Pirofski (2007), soil is an extreme environment with rapidly changing conditions, and bacteria living in soils will encounter an enormous number of predators of different types: unicellular amoebas, slime molds, protists, nematodes, snails as well as larger animals. As they are likely to meet ever-changing conditions as well as predatory hosts with different types of receptors and antimicrobial defenses, soil dwellers have to carry a diverse array of characteristics including host cell adhesins and immune modulatory molecules as defense mechanisms against predators. It was beyond the scope of our study to identify the bacterial factors involved in adhesion. However, a computational and experimental analysis of the *M. capsulatus* secretome has previously identified *M. capsulatus* Bath protein homologs of adhesion proteins that are involved in microbe adhesion to host cells in other bacterial species (Indrelid et al., 2014), showing that candidate host interaction proteins are present in *M. capsulatus* Bath and may facilitate adhesion to DC.

The maturation state and cytokine profile of DCs is functionally important. Although DCs represent a heterogeneous group of antigen-presenting cells, they have traditionally been divided into immature and mature differentiation stages (Reis e Sousa, 2006). Immature DCs are characterized by low surface expression of major histocompatibility complex (MHC) class II molecules and co-stimulatory molecules (e.g., CD80, CD86, and CD40). However, when DCs encounter microbes, pattern-recognition receptors (PRRs) are triggered by microbe-associated molecular patterns resulting in major changes in gene expression and acquisition of a number of functional properties: antigen processing and presentation, migration, and T-cell co-stimulation (Dalod et al., 2014).

It has been proposed that pathogen, probiotic, and commensal bacteria can be divided into three distinct classes based on the extent of host response by DCs and other PRR expressing cells. MAMPs of pathogenic microorganisms tend to induce a strong host response, probiotics induce an intermediate response whereas commensal bacteria exhibit homeostatic control of the response (Lebeer et al., 2010). In the present study the non-commensal, non-pathogenic *M. capsulatus* Bath induced a DC response intermediate between the Gram-positive probiotic *Lactobacillus rhamnosus* GG and the commensal Gram-negative *E. coli* K12. Substantial differences were found between *M. capsulatus* Bath, *L. rhamnosus* GG and the *E. coli* K12 in their ability to induce phenotypical and functional maturation of monocyte-derived DCs. Toll like receptor 4 is expressed on MoDCs and recognize lipopolysaccharide (LPS), the major component of the outer membrane of Gram-negative bacteria (Schreibelt et al., 2010). LPS represents a strong stimulatory signal for inducing expression of co-stimulatory molecules and cytokine production in DCs (Verhasselt et al., 1997). Concordantly, *E. coli* K12 and *M. capsulatus* Bath were found to be stronger inducers of DC maturation and cytokine release compared to the Gram-positive *L. rhamnosus*. It has been suggested that probiotic bacteria modulate immune response by controlling the maturation of DCs and thereby the release of proinflammatory cytokines (Foligne et al., 2007). Both the Gram-negative bacteria tested in our study induced phenotypical and functional maturation. However, *M. capsulatus* Bath produced a less mature phenotype and substantially lower cytokine levels than *E. coli* K12. The fact that the Gram-negative *M. capsulatus* Bath results in a less mature phenotype and low levels of proinflammatory cytokines, suggests that similarly to probiotic bacteria *M. capsulatus* may modulate immunity through directing the maturation of DCs.

Interestingly, although priming with *M. capsulatus* resulted in a less mature MoDC phenotype than *E. coli* K12, it was found more efficient than both *E. coli* K12 and *L. rhamnosus* GG bacteria in inducing T cell activation and proliferation in the presence of interleukin 2, a growth factor necessary for cell cycle progression and clonal expansion (Smith, 1988). *M. capsulatus*-primed MoDCs enhanced T cell expression of CD25, the α-chain of the trimeric high affinity IL-2 receptor explaining the increased proliferative T cell response compared to the other bacteria.

Whereas, co-stimulatory molecules on DCs and T cells are necessary for T cell activation and proliferation, DC cytokines are central in polarizing effector T cell development. In addition to antigen presentation and signaling through co-stimulatory molecules, cytokines provide a third signal for activation and differentiation of naïve T cells to effector cells. The nature of signal 3 depends on the triggering of particular PRRs by MAMPs specific to the microbe encountered (Kapsenberg, 2003). Several DC-derived cytokines are important for T cell polarization into specific T cell subsets, e.g., IFNγ and IL-12p70 are known to be important for Th1 polarization, IL-4 is essential for the Th2 differentiation process, and TGF-β to TH17 and Tregs. Although *M. capsulatus* behaved more similar to *E. coli* than *L. rhamnosus* in its ability to induce cytokine production from MoDCs, both the magnitude and cytokine profiles of the two strains were different. Both strains for example induced similar levels of IL-2, but *E. coli* induced considerably higher levels of IL-23, a cytokine linked to the generation and maintenance of Th17 functions. *M. capsulatus* induced negligible IL-1β, and compared to *E. coli* substantially less of Th1 polarizing factors IL12p70 and IFN-γ as well as reduced IL-6 levels. IL-6 is a cytokine that plays a role in proliferation and survival of both Th1 and Th2 cells, is important for the commitment of CD4^+^ cells to the Th17 and Th22 lineages and has an inhibitory role in Treg development (Hunter and Jones, 2015).

Bacteria-induced differences in MoDC cytokine production were also functionally reflected in different T cell polarizing ability in MoDC-T cell co-cultures. In response to bacteria-primed MoDCs, T cells produced increased levels of the anti-inflammatory cytokine IL-10. IL-10 plays important roles both in preventing inflammatory responses to intestinal microbiota under steady state conditions, and in limiting T cell-driven inflammation in pathogen clearance (Maynard and Weaver, 2008). Notably, MoDC-priming with all three bacteria significantly increased concentration of the Th1 signature cytokine IFN-γ and reduced the levels of typical Th2 cytokines IL-13 and IL-5. Th2 development has previously been suggested to be a “default pathway” in the absence of IL-12 (Moser and Murphy, 2000). In agreement with that, in our experiments unprimed MoDCs tended to induce Th2 cell responses compared to MoDCs primed by bacteria. The observation that even the Gram-positive *L. rhamnosus* drives Th1 development suggest that LPS is not a critical factor in bacteria driven DC-mediated Th1 development, in support of previous reports (Smits et al., 2004).

Coherent with results from DC cytokine analysis, the cytokine profile of T cells co-incubated with MoDCs primed by Gram-negative bacteria was markedly different from that of T cells activated by MoDCs treated with the Gram-positive *L. rhamnosus*. Again *L. rhamnosus* resulted in lower levels of most of the cytokines measured, a reduction in the pro-inflammatory IL-18 and no increase of IL-1α, IL1-β, IL-6 compared to negative control. Neither did it induce cytokines typically associated with Th17/Th9/Th22 cells (IL-23, IL-17A, IL-21, IL-22, IL-9, TNF-α) compared to a control of T cells stimulated by unprimed DC. The low T cell-levels of cytokines in response to *L. rhamnosus* is in agreement with a previous report showing that *L. rhamnosus*-primed MoDCs induce hyporesponsive T cells in DC-T cell co-cultures (Braat et al., 2004).

In contrast to *L. rhamnosus M. capsulatus* Bath, and *E. coli* K12 induced proinflammatory cytokines IL-6, IL-1β, and IL-1α as well as cytokines associated with generation and maintenance of the Th17 subset (IL-23, IL-17A, IL-21, IL-22), Th22 cytokines (IL-22, TNF-α) and Th9 cytokines (IL-9 and IL-21). However, *M. capsulatus* induced significantly less pro-inflammatory cytokines IL1-α, IL-1β, and IL-6 and anti-inflammatory IL-10. There was no significant difference in the Th1 signature cytokine IFN-γ or Th9 cytokines IL-9 and IL-21. However, significantly less IL-23, IL-17A, and IL-22 was produced in response to *M. capsulatus* than to *E. coli*. The cytokine profile thus indicated that different effector cells dominate in response to the two Gram-negative bacteria. *E. coli* is a stronger inducer of the Th17 subset whereas *M. capsulatus* induce Th1/T9 effector cells over Th17 cells *in vitro*. Some probiotics have been reported to induce Foxp3+ regulatory T cells (Kwon et al., 2010). It has been suggested that peripherally-induced Treg develop from naïve, CD4+ cells exposed to antigens under tolerogenic conditions (e.g., by immature DCs with low levels of co-stimulation) with an essential requirement for TGF-β signaling (Marie et al., 2005; Johnston et al., 2016). We did not find detectable levels of TGF-β released from MoDC stimulated by *M. capsulatus*. Neither did we find significantly increased expression of Foxp3 in T cell co-cultures with bacteria stimulated MoDC (data not shown).

*E. coli* and *L. rhamnosus*, but not *M. capsulatus* Bath, reduced lymphotoxin-α and IL-1RA in culture supernatants. Lymphotoxin-α is important for lymphoid organ development, regulates T cell homing and IgA production in the gut and contributes to shaping the gut microbiome (Ruddle, 2014). The balance between IL-1 and IL-1RA in local tissues plays an important role in the susceptibility and severity of a number of diseases, including IBD (Arend, 2002). For example, significant decrease in the intestinal mucosal IL-1RA/IL-1 ratio has been found in freshly isolated intestinal mucosal cells, and in mucosal biopsies obtained from both Crohn's disease and ulcerative colitis patients as compared to control subjects (Casini-Raggi et al., 1995). The observation that IL-1α and IL-1β is reduced, while IL-1RA is kept high in *M. capsulatus* primed DC-T cell co-cultures is interesting in the light of *M. capsulatus* anti-inflammatory effects in a murine enteritis model (Kleiveland et al., 2013). Screening for cytokine profiles associated with specific T effector cell populations may be a useful first step to identify strains with potential pro- or anti-inflammatory properties e.g., for further mechanistic investigation (Papadimitriou et al., 2015). It is important however to notice the limitations of *in vitro* systems on making *in vivo* predictions. Although the bacteria tested here induced different effects in T cells *in vitro*, caution should be exercised in drawing conclusions both about the direction of T cell polarization by these bacteria and the functional relevance *in vivo*. T cell differentiation occurs in a finely tuned manner dependent on a variety of tissue factors and cytokines, and *in vitro* systems cannot necessarily reflect the complex cytokine environment of the gut. For example, TGF-β a cytokine abundant in the intestines, was not detected in our MoDC supernatants. TGF-β is not only involved in development of Tregs, Th9 and Th17 effector cells, but it also suppresses Th1 and Th2 cell differentiation (Zheng, 2013). TGF-β is produced by CD103^+^ DC (Coombes et al., 2007) a DC subset common in the intestines and is expected to play a prominent role in regulating mucosal immunity (Ruane and Lavelle, 2011). The results of bacterial priming *in vitro* may thus be expected to have different outcomes in an *in vivo* situation. The impact of immune modulatory effects of *M. capsulatus* on DC in maintaining intestinal homeostasis thus remains to be determined (study in preparation).

## Concluding remarks

Environmental bacteria, although immensely numerous and diverse, have remained largely unexplored for their immunomodulatory properties. Our results demonstrate the direct binding and functional effects of a soil bacterium on human monocyte-derived dendritic cells. The same bacterium has recently been shown to possess anti-inflammatory properties in a murine colitis model. The identification of anti-inflammatory and immunomodulatory properties of this bacterium was serendipitous. In fact, such properties may not be a rare trait of this particular soil bacterium, but rather a common feature of many environmental bacteria. Our study thus emphasizes the need to scrutinize, identify, and understand possible physiological consequences of environmental microbe-host interactions, and we suggests that bacteria from soil and water should receive increased attention for their potential health benefits.

## Author contributions

SI contributed to design of the work, acquisition, analysis, and interpretation of data and drafted the work. TL and CK contributed to design of the work, interpretation of data and revising work critically for important intellectual content. RH contributed to data analysis and revising work critically for important intellectual content. MJ contributed to interpretation of data and revising work critically for important intellectual content. All authors approved final version and agreed to be accountable for all aspects of the work.

### Conflict of interest statement

The authors declare that the research was conducted in the absence of any commercial or financial relationships that could be construed as a potential conflict of interest.
